# Magic and Misdirection: The Influence of Social Cues on the Allocation of Visual Attention While Watching a Cups-and-Balls Routine

**DOI:** 10.3389/fpsyg.2016.00761

**Published:** 2016-05-31

**Authors:** Andreas Hergovich, Bernhard Oberfichtner

**Affiliations:** Department of Applied Psychology: Work, Education and Economy, Faculty of Psychology, University of ViennaVienna, Austria

**Keywords:** magic, eye-tracking, social misdirection, inattentional blindness, joint attention, amazement

## Abstract

In recent years, a body of research that regards the scientific study of magic performances as a promising method of investigating psychological phenomena in an ecologically valid setting has emerged. Seemingly contradictory findings concerning the ability of social cues to strengthen a magic trick’s effectiveness have been published. In this experiment, an effort was made to disentangle the unique influence of different social and physical triggers of attentional misdirection on observers’ overt and covert attention. The ability of 120 participants to detect the mechanism of a cups-and-balls trick was assessed, and their visual fixations were recorded using an eye-tracker while they were watching the routine. All the investigated techniques of misdirection, including sole usage of social cues, were shown to increase the probability of missing the trick mechanism. Depending on the technique of misdirection used, very different gaze patterns were observed. A combination of social and physical techniques of misdirection influenced participants’ overt attention most effectively.

## Introduction

### Looking Back on the Science of Magic (and Misdirection)

Several years after publication of the article “Towards a Science of Magic” (Kuhn et al., 2008a), in which the authors argued for the scientific exploitation of the audience-proven methods of professional stage magicians, a considerable amount of research has been conducted to elucidate some of the mechanisms behind the illusions magicians have been developing and refining over decades and centuries. Although today a great variety of research approaches exist among the contributions to the field (for an overview, see Rensink and Kuhn, 2015; Thomas et al., 2015), a good part of the research conducted in the name of “science of magic” put its focus on the study of the diversion and deflection of peoples’ (visual) attention to conceal the mechanism of a trick (i.e., attentional misdirection; Kuhn et al., 2014).

What are the mechanisms through which peoples’ attention can be orchestrated in a way that increases their susceptibility to deliberate deception? Kuhn and Tatler (2005) were the first to try to answer this question by examining the eye movements of observers of a magic trick (Tatler et al., 2014). During the performance of a self-invented live routine, Kuhn and Tatler (2005) seemingly let a cigarette vanish. Only two out of 20 participants discovered the trick mechanism after watching the routine for the first time. All of them discovered it after the second time.

Surprisingly, Kuhn and Tatler (2005) found little difference in gaze deployment between deceived and undeceived individuals, or the first and second viewing of the trick, suggesting the misdirection of covert rather than overt attention. Overt attentional orienting happens by directing one’s eyes toward a stimulus for optimal information processing, which can be observed externally. Covert orienting happens by shifting one’s attentional spotlight without corresponding body movements and has to be inferred (Posner, 1980), for example, by measuring whether a specific event was detected.

Because the cigarette was fully visible while being dropped into the lap of the performer, Kuhn and Tatler (2005) likened the observers’ inability to perceive the trick mechanism to inattentional blindness, a phenomenon probably best exemplified by the widely known “invisible gorilla” experiment (Simons and Chabris, 1999). Although there is some debate about the degree of similarity between attentional misdirection and inattentional blindness (Memmert, 2010; Kuhn and Tatler, 2011), both terms are often used interchangeably (e.g., Barnhart and Goldinger, 2014). Memmert (2006) found a decoupling of overt and covert attention using the “invisible gorilla” paradigm.

In their “Psychologically based taxonomy of misdirection”, Kuhn et al. (2014) differentiate between several internal and external triggers of attentional misdirection. Kuhn and Tatler (2005), however, could make no assertions regarding the relative effectiveness of each of the potential triggers occurring during their routine. Misdirection by visually salient movements (i.e., physical misdirection), by surprise, or by the performers social cues are all plausible explanations.

### The Role of Social Cues

Magic performances are inherently social situations. Social cues occupy a special place in the literature on visual attention. Attentional orientation elicited by social cues, especially the position of the eyes and head, has been shown to be more reflexive and involuntary than other kinds of cues when presented in the center of peoples’ visual field (for an overview, see Frischen et al., 2007). The usage of life-like material is, however, rather uncommon in the research on social attention, potentially hampering the transfer of its insights to the realm of magic and real-life human interaction.

Perhaps the first experiment to show enhancement of a magic illusion purely by use of social cues was reported by Kuhn and Land (2006). In the “vanishing ball illusion,” a magician throws a ball into the air several times and catches it, but the last throw is only pretended; the magician keeps the ball hidden in his hand. Viewers typically perceive the ball as being thrown upward and vanishing midflight. When the magician followed the presumed movement of the ball with his eyes and head, participants succumbed to the illusion more readily than when the magician kept his gaze directed toward his hand. In this instance, social cues, rather than concealing the vanishing of an object, reinforced the illusory perception of an object.

Subsequent variations on the original experiment by Kuhn and Tatler (2005) have strengthened the notion of the importance of social cues in orchestrating attention. Kuhn et al. (2009) used two versions of a trick in which a lighter was dropped: one in which a misdirecting arm movement was supported by the magician’s head movement and one in which the gaze of the magician remained directed toward the location of the dropping lighter. Both covert and overt attention were influenced by the social cue. Participants were deceived more readily when the social cue was congruent with the arm movement and spent less time looking at the area of the trick mechanism. Nevertheless, the capacity for attentional misdirection of pure social cues was not demonstrated in this experiment as additional triggers occurred in both conditions.

Kuhn and Findlay (2010) further found that visual fixations and deception rates were associated in a temporally flexible way and that covert attention seems to prepare subsequent eye movements. Using video-editing techniques, which made perceiving the trick mechanism impossible, they were also able to demonstrate that participants’ verbal reports were representative of their actual perceptions rather than false memories or inferences about the trick mechanism.

### Doubts about Social Cues

The relevance of social cues for the outcome of magic tricks has, however, been called into question by Cui et al. (2011) and Rieiro et al. (2013). Renowned stage magicians contributed to both studies.

Cui et al. (2011) investigated the influence of several variables on the illusory perception of a tossed coin. One of these variables was the employment of a social cue. Just as in Kuhn and Land’s (2006) experiment, the social cue was not intended to divert attention away from an act or object but to enhance perception of an illusory movement.

In one of the experiments on which they report, participants were shown a series of 100 videos. In each video, the magician either tossed a coin from one hand to the other or only pretended to, keeping the coin hidden in his hand. Slight variations were made to this basic choreography, but in all of them, the magician made eye contact with the viewers at the moment of the (supposed) coin toss. Half of the videos were edited so that the magician’s face and shoulders were occluded by a black rectangle. Because participants did not report more coin tosses when the magician’s face was visible, the authors concluded the employment of the social cue to be ineffective.

Regarding the conclusion of Cui et al. (2011), it should be noted that during the presentation of an actual magic trick the alternative to making eye contact is hardly ever occlusion of the performer’s face but simply making no eye contact. This disparity severely reduces ecological validity, which is otherwise a hallmark of the science of magic. Of course, the difficulty of performing the same trick twice in exactly the same manner is formidable, which makes resorting to video manipulation an attractive option. However, covering about 17.5% (own calculation) of the image area with a large black rectangle seems to create more problems than it solves. It could be argued that the determinant in the comparison group was not the absence of a social cue but the presence of a large black rectangle. In our own experiment, we have tried to create a more natural control condition.

The main rationale of Cui et al. (2011) for occluding the face area was the creation of a “socially neutral” condition. They remarked that in Kuhn and Land’s (2006) experiment, the magician either supported the illusory ball movement with his head movements or looked at the actual position of the ball. However, in the experiment of Cui et al. (2011), the illusory coin toss, at the moment it ostensibly happened, was not supported in any of the conditions; instead, eye contact was made.

Cui et al. (2011) assumed that viewers who reciprocate the gaze of the performer view the trick peripherally. They further reasoned that the increased responsiveness of peripheral receptive fields to actual motion would also make perceiving the illusory movement more probable. Just the opposite was the case: viewers reported a higher number of coin tosses when the performer’s face was occluded.

This should not be surprising, as to our knowledge magicians report this trick to be more effective when spectators are fully attentive to the performer’s hand movements. Regardless of the motion sensitivity of peripheral vision, motion perception itself is probably not sufficient to create the illusion of the toss of a specific object like a coin.

We assume social cues to be most effective in strengthening an illusory motion when they are congruent with the performer’s other movements (e.g., Kuhn and Land, 2006). On the other hand, usage of social cues to divert attention away from an event (e.g., Kuhn and Tatler, 2005) should be regarded as a different phenomenon altogether. We think the magician has to make a fundamental decision about the desired effect. Should the social cue suggest an event that did not occur (like a coin toss) or distract from an actual event? It may not be possible to achieve both at the same time.

Lateral gaze cues have been shown to cause reflexive visuospatial orienting of covert (Friesen and Kingstone, 1998), as well as overt (Mansfield et al., 2003), attention. Direct eye contact can delay disengagement of visuospatial attention as well (Senju and Hasegawa, 2005). However, these two ocular social cues are processed rather differently (Pfeiffer et al., 2013). In our experiment, we therefore consider lateral gaze cues and eye contact separately.

For participants, learning to differentiate correctly between real and fake tosses was rather difficult, which suggests that this task approaches fundamental perceptual limitations (e.g., Hergovich et al., 2011). Cui et al. (2011) themselves note that the trick was performed with great manual dexterity and that in such cases enhancement by additional attempts of misdirection may not be possible (see Phillips et al., 2015). Therefore, magic tricks used to examine the influence of a particular trigger of misdirection should provide the viewer with a fair chance of being undeceived in the absence of the trigger. In studies using an inattentional blindness paradigm, the ability of participants to detect the relevant stimulus in a control condition is a prerequisite (Memmert, 2010).

### The Cups and Balls

Rieiro et al. (2013) explored, among many other factors, the role of social cues and came to similarly negative conclusions. In this study, a special variant of a cups-and-balls routine popularized by the entertainment duo Penn and Teller was used. The trick relies on sleight of hand to surreptitiously introduce a ball inside an upside-down cup while the movement of another ball dropping from its top misdirects viewers’ attention. This dropping ball is so attentionally compelling that the appeal of the trick persists even when viewers are fully informed about its mechanism. Although no active attempt of social misdirection was made, the effect of occlusion of the performer’s face by a black rectangle was assessed. No change in trick effectiveness through face occlusion was found. However, we do not agree with the authors’ conclusion that “the ‘Cups and balls’ magic trick does not rely on social misdirection” (p. 11). Our current experiment suggests that different trick choreographies may vary strongly in their dependence on attentional misdirection.

Tachibana and Kawabata (2014) took up the paradigm of the cups-and-balls routine and, using their own version of the trick, explored the effect of different spatial positions of misdirection. They found that, while misdirection positions at the back of the table were viewed longer than were those at the front, whether misdirection happened on the left side, on the right side, or on the center of the table made no difference. When visual fixations of deceived and undeceived individuals were compared, it was observed that deceived individuals spent more time looking at the performer’s face than undeceived individuals.

However, whether Tachibana and Kawabata’s (2014, p. 143) conclusion that “deceived individuals have difficulty trying not to allocate attention to the face” is cogent is debatable. After all, those participants who, by chance, happened to look at the area of the head at the time of the trick event may have simply been less likely to perceive the trick mechanism. This possibility would not imply any difficulty not to allocate attention to the head or even a preference for doing so. In addition, even if deceived individuals were more responsive to social cues, it would not necessarily follow that they would attend to the head longer in the absence of such cues.

More generally, the design of the experiment, randomized within-subject presentation of 12 unique videos and comparison of these videos among 20 subjects, leaves unanswered questions. It is unclear, for example, when the eventually undeceived participants discovered the trick mechanism. If they did so late in the experiment, then they too were deceived during most of the presentations. In any case, order and sequence effects cannot be ruled out. To prevent this, single presentations to a much larger number of participants would have been preferable.

The relative influence of social cues on viewers’ covert and overt attention could not be examined in the course of this experiment because it lacked control conditions in which no attempts of social misdirection are made. In addition, the influence of the performer’s cues could not be considered in isolation because in each video, it was confounded by the simultaneous revelation of a visually salient red ball at the position emphasized by the performer’s gaze.

### Our Approach

What can be concluded about the role of social cues in magic performances? Can social cues indeed be used to enhance magic tricks, as many magic practitioners intuitively assume? If yes, can they be effective on their own or only if used to supplement physical triggers of misdirection? Or can the negative findings of Cui et al. (2011) and Rieiro et al. (2013) be generalized to many other kinds of trick choreographies? To answer these questions and to caution against such generalizations, we devised a cups-and-balls routine in which social cues have a realistic chance of playing a strong role.

We attempted to disentangle the effects of several triggers of misdirection that were confounded in previous research (making eye contact, lateral misdirection by sideward gaze, lateral misdirection by a visually salient stimulus, and the combination of sideward gaze with a visually salient stimulus) and tried to make them mutually comparable. In addition, an unedited socially neutral control condition was included to satisfy a common point of criticism (e.g., Memmert, 2010). In our routine, only two cups were used, none of which was preferentially looked on in the control condition or when eye contact was made.

Participants’ gaze positions while watching the routine were recorded using an eye-tracker as a measure of overt visual attention. An open question about the trick mechanism served as a measure of covert attention at the moment of the trick event (see Kuhn and Findlay, 2010). To allow unambiguous interpretation of the effects of the five experimental conditions, each participant was exposed to only one kind of trick choreography in a between-subjects design.

To assess the stability of misdirection effects and to explore changes in gaze deployment, the same choreography was presented twice to each subject. To maximize ecological validity, the video recordings participants were presented with had been recorded in one take, without cuts or edits altering the actual sequence of events. Although this approach may offer interesting insights into general aspects of attentional orienting in natural scenes, we do not make any claims of ecological validity outside the realm of magic. Certainly, for most people, watching a magic performance (or videos thereof) is a unique situation and it is the aim of this study to investigate whether certain techniques of misdirection are effective in this specific context.

To facilitate analysis of gaze positions and to allow for effective misdirection, most of the available image area was utilized. The head of the performer, the area of misdirection, and the area in which the trick event took place were spatially distinct, and the movements of the performer were expansive and clearly observable.

Only the initial presentation of the trick was expected to be informative regarding the effectiveness of social misdirection. Therefore, to compare how deceived and undeceived participants differ in their reaction to social cues, achieving a balanced distribution of deceived and undeceived individuals after only one presentation (which by the standards of stage magic would otherwise be a fairly unsatisfactory outcome) was deemed desirable. Because Tachibana and Kawabata (2014) reported 55% of participants to be deceived after six presentations of their version of the routine, the mechanism of the trick needed to be considerably more transparent.

To this end, several variations of the choreography expected to be most effective in misdirecting viewers’ attention (the combination of physical and social triggers) were pretested until we were confident that the trick mechanism would be sufficiently easy to detect while not being immediately obvious. The trick itself (the loading of a ball into a cup) was performed in an unorthodox fashion. The ball was clearly visible for a fraction of a second to ensure detection if visual attention is appropriately deployed. Successful deception of participants was dependent on them being inattentionally blind at the moment of the trick.

Because the effectiveness of gaze cues can differ depending on the visual field they are presented to (Okada et al., 2006), the position of the performer’s head remained central. Conversely, the lateral alignment of a trick event has not been found to influence its effectiveness (Barnhart and Goldinger, 2014; Tachibana and Kawabata, 2014), which is why the trick also happened at the same place (the right side of the participants’ field of view) in all conditions.

Following Rensink and Kuhn’s (2015) suggestion to explore individual differences in the perception and judgment of magic tricks, this experiment was designed to balance male and female participants within each condition so that possible sex differences could be assessed. In accordance with findings of research using a gaze-cueing paradigm (Bayliss et al., 2005), male participants were expected to be less reactive to attempts of social misdirection.

Consistent with gaze cueing research (Frischen et al., 2007) participants were expected to orient covert attention toward the area of lateral misdirection more strongly in those conditions in which an attempt of lateral social misdirection was made. In comparison with the control condition, this would result in an increase in the number people not being able to identify the trick mechanism correctly. This would also confirm the intuitions of illusionists about the effectiveness of social cues and refute the suggestion that they may not play a significant role in cups-and-balls routines (Rieiro et al., 2013).

In accordance with fundamental research on attentional orienting (e.g., Posner, 1980) participants were also expected to more strongly orient covert attention toward the area of lateral misdirection in those conditions in which an attempt of lateral physical misdirection was made. This would suggest that in cases where both strategies are combined (e.g., Kuhn and Tatler, 2005), social and physical triggers both exert an effect on viewers’ attention.

Participants were further expected to covertly reciprocate eye contact (e.g., Senju and Hasegawa, 2005), increasing deception rates and thereby refuting the assumption of Cui et al. (2011) that making eye contact would enhance motion perception in the periphery.

All forms of misdirection were hypothesized to also affect overt attention within a specified time frame. This would suggest a coupling of covert and overt attention (i.e., missing the trick mechanism and gaze direction) and confirm recent research by Kuhn and Findlay (2010).

Deceived individuals, we predicted, would direct more of their overt attention to the performer’s head, whether social misdirection is employed or not. Tachibana and Kawabata (2014) already hinted at this possibility but could not conclusively demonstrate it.

As in previous experiments (e.g., Tatler and Kuhn, 2007), deception rates were expected to be significantly lower during the second presentation of the routine. We believed this to be caused by a change in participants’ viewing strategy. We wanted to explore the nature of this change in strategy by comparing the relevant eye tracking data.

We also explored the subjective qualities of experiencing a magic performance, by supplementing the mere recording of deception rates and eye tracking data with a suitable questionnaire. Hergovich (2004) used an inventory consisting of 12 questions to let participants assess their sensations after having witnessed a pseudo-psychic demonstration. We reused this inventory here to investigate how participates’ assessment of the cups-and-balls routine would vary depending on the experimental condition and their ability to identify the trick mechanism.

## Materials and Methods

### Participants

From the students and staff of the University of Vienna, 120 participants (60 female, 60 male) were recruited and took part voluntarily without any kind of compensation. Sixty-four of them (53%) were psychology students. The average age was 25.38 years (*SD* = 5.43) with a range from 19 to 52 years. Twelve participants (10%) were left-handed. All participants had normal or corrected-to-normal eyesight. For all participants, horizontal and vertical gaze deviation did not exceed 0.9° visual angle during the initial calibration procedure of the eye-tracker. Ten additional subjects were recruited but could not take part in the experiment because the calibration procedure yielded unsatisfactory results. Only one participant reported having difficulties distinguishing between the colors red and green. However, in the experimental condition to which he was (randomly) assigned, neither green nor red stimuli were presented.

### Materials

Ten videos in which the second author performs a cups-and-balls routine were created. To reduce the relative influence of idiosyncrasies of the video material or involuntary movements by the performer that had nothing to do with the experimental manipulation on the results, two videos were created for each experimental condition that were designed to be as similar as possible but represented different performances recorded at different times. Each participant was randomly assigned to see one of these videos and saw none of the other nine during the experiment. When combined in pairs, videos with identical choreographies formed the five experimental conditions.

Each video had a total duration of 36.6 s. The movements of the performer had been carefully timed using a metronome, to maximize between group comparability. Each major movement of the performer, as well as the pauses in between, took 4 beats of the metronome for 48 beats per video. All choreographies were the same, except for the events between seconds 18 and 21 of the video, in which the trick event and, depending on the experimental condition, an attempt of misdirection of the viewers’ visual attention happened.

**FIGURE 1:**
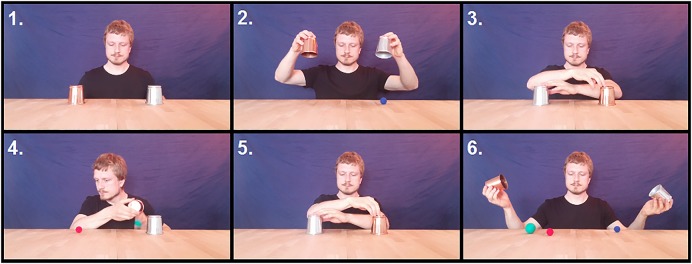
**Sequence of events that are part of the choreography presented in the videos.** The beginning of the video (1). The blue ball is shown (2). The cups are shuﬄed (3). The green ball is loaded into the cup (4). The cups are shuﬄed again (5). The cups are lifted and all balls are revealed (6). All events shown here, except events 4 and 6, are alike in all videos. A depiction of all the different versions of event 4 is shown in **Figure 2**.

The videos show the performer sitting behind a wooden table on which one bronze colored cup (on the left side of the screen) and one silver colored cup (one the right side of the screen) are placed (see **Figure 1**). The background is covered with navy blue drapery. Throughout the act, the gaze of the performer remains directed toward the middle of the table. First, both cups are lifted, and a small blue ball, which was hidden under the silver-colored cup, is revealed. Then both cups are placed on the table as they were before and are shuﬄed by simultaneously moving each cup to the other side of the table and back again. Now the performer lifts the bronze-colored cup (on the left side of the screen) with his right hand and moves the cup toward the left side of his upper body (the right side of the screen). While the opening of the cup is oriented toward the performer, he grabs a relatively large, bright green ball, which was hidden beneath the table, with his left hand and quickly places (loads) it inside the bronze-colored cup. He does so making no effort to conceal the ball, orienting the front side of his hand toward the observers. The cup is then placed in its original position in such a way that the green ball remains hidden beneath it.

**FIGURE 2:**
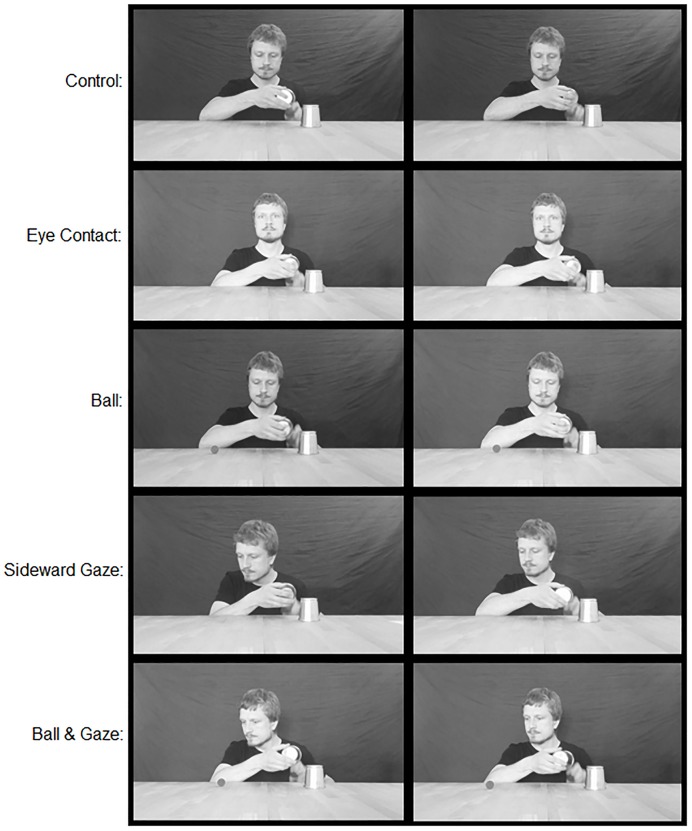
**Depiction of the moment of the loading of the green ball (i.e., the trick mechanism to be detected) in all of the videos used in the experiment.** Each row represents a different experimental condition. The two images in each row show the critical event in both versions of the videos that were combined to form an experimental condition.

During the time the loading of the green ball takes place, the videos corresponding to the five experimental conditions differ regarding the additional actions of the performer (see **Figure 2**):

(1) Control Group: In the videos shown to the control group, no attempt of misdirection of the viewers’ attention is made. The gaze of the performer remains directed toward the middle of the table for the entire duration of the video.
(2) Eye Contact: The performer raises his gaze from the table the moment he begins lifting the bronze-colored cup, and looks toward the camera, thereby creating eye contact with the viewer. He breaks eye contact and returns his gaze toward the table the moment the bronze-colored cup is returned to its original position.
(3) Red Ball: The gaze of the performer remains directed toward the middle of the table for the entire duration of the video. When the performer lifts the bronze-colored cup, a red ball appears beneath it. The ball is of the same size as the blue ball revealed to be under the silver-colored cup at the beginning of the video. During the first half of the video, it remained attached to the bottom of the bronze-colored cup by a magnetic mechanism and is only now surprisingly revealed. It remains visible during the loading phase until the bronze-colored cup, which now also contains the larger green ball, is placed above it again.
(4) Sideward Gaze: Shortly after the bronze colored cup is lifted, the performer turns his head to his right (the left side of the screen), leans slightly forward, and, with his eyes fully opened, directs his gaze toward the previous position of the cup. The shift of the performer’s gaze direction is the only method of misdirection employed. After the cup is returned to its original position, the performer directs his gaze back toward the middle of the table and assumes his original posture.
(5) Ball & Gaze: This condition simply represents a combination of the Red Ball and Sideward Gaze conditions.

The videos were recorded at a rate of 29.75 frames per second and at a resolution of 1920 pixels × 1080 pixels. During the loading of the green ball, it is clearly visible in full size for the duration of three to four single frames (100–134 ms). For presentation in the laboratory, the videos were re-encoded at twice the frame rate and scaled to fit the aspect ratio of the computer monitor used.

The material was presented on a Dell P2210 22″ LCD monitor (1920 × 1050, 60 Hz), connected to the ATI Mobility Radeon HD 4500 Series graphics card of a laptop computer (2.80 GHz, 3 GB RAM), at a viewing distance of 60–80 cm. The programming of the experiment, as well as the collection and processing of the gaze data, was accomplished using the SMI Experiment Suite 360 software bundle. Gaze data were collected binocularly using an SMI RED 500 eye-tracking system at a spatial resolution of 0.03° visual angle and a sampling rate of 250 Hz.

In addition to questions gathering basic demographic data and several questions pertaining to the mechanism of the trick, a questionnaire, consisting of 12 items, used by Hergovich (2004) to assess a deception demonstration, was presented to participants. Although the questionnaire was originally designed to gauge the reactions of participants after having watched a supposed act of telepathy, it was used in this context to measure, among other factors, “amazement” of participants after watching the cups-and-balls routine.

We also asked participants to rate the degree of previous experience with magic tricks (on a one-to-seven point Likert scale), to control for the possibility of it being a confounding factor (e.g., Demacheva et al., 2012).

### Procedure

Participants were asked individually and personally to participate in an eye-tracking study about magic tricks. After agreeing to participate, they were led to the laboratory, where written informed consent was obtained. Then the room was darkened, and the calibration procedure of the eye-tracker was started. Participants were only allowed to continue if gaze deviation was less than a 0.9° visual angle. If the participant’s gaze deviation was greater at the first attempt, the calibration procedure was repeated a maximum number of four times.

At the beginning of the experiment, participants were randomly assigned to watch one of the ten videos described earlier. Assignment occurred using a random number generator in such a way that each video could be seen by 12 people (six males and six females). After reaching the desired amount of participants per video, randomized assignment occurred only to the remaining ones.

Once the experimental session began, all participants answered demographical questions and were then informed that they would see a video of a magic trick. They were instructed to watch the upcoming events closely but were not told that they would have to answer questions pertaining to the trick mechanism. After looking at a fixation cross in the middle of the screen for a duration of 2 s, playback of the video assigned to them started, during which eye-tracking data were recorded.

After having watched the video, they were asked the following question: “In the video you just saw, a large, green ball appeared under one of the cups. Please describe briefly how you think the ball got there,” which they were able to answer freely using their own words. They were then asked if they actually saw what they just described or if they just suspected that their explanation could be correct. They had to choose between these two options. In addition, they were asked to rate (on a one-to-seven point Likert scale) how confident they were that the ball got under the cup in the exact way they described.

They then were shown the same video a second time, and all the questions pertaining to the trick mechanism were repeated. Finally, they answered the questionnaire for the assessment of a deception demonstration (Hergovich, 2004) and gauged their own prior experience with magic tricks.

## Results

### Deception Rates

Two judges dichotomously categorized the participants’ explanations of the trick mechanism according to whether they correctly identified the mechanism or did not. The judges independently categorized two statements by each of the 120 participants: one given after the first presentation of the magic trick and one given after the second presentation. Disagreements between the judges were resolved through discussion. Interrater agreement was high, with four disagreements for the first set of statements (Cohen’s κ = 0.93) and two disagreements for the second set of statements (Cohen’s κ = 0.92).

An example of a participant’s answer that was categorized as “undeceived” is “The ‘magician’ put it in the cup as he briefly lifted it (the opening oriented toward himself).” An example of a participant’s answer that was categorized as “deceived” is “It is a video manipulation for which the video was stopped, so the green ball could be added.” An example of a participant’s answer that initially caused disagreement and was categorized as “deceived” after discussion is “First in the hand—it went into the cup later.”

After the first presentation 4 (7.7%) of the 52 participants whose answers were categorized as “deceived” stated to have seen what they described as the trick mechanism whereas 45 (66.2%) of the 68 “undeceived” participants have done so, χ^2^_(1, *N* = 120)_ = 41.72, *p* < 0.001. After the second presentation, 5 (33.3%) of the 15 participants who remained “deceived” stated to have seen the trick mechanism while 94 (89.5%) of the 105 “undeceived” participants have done so, χ^2^_(1, *N* = 120)_ = 24.94, *p* < 0.001 (Yates’s correction for continuity was applied because of low expected frequency).

The ratings of the judges and the participants’ responses to the question of whether they “saw” what they wrote down or just “suspected” it were found to be substantially, but not strongly, correlated (φ = 0.590, *p* < 0.001). This finding seemingly contradicts the findings of Kuhn and Findlay (2010) concerning the validity of participants’ statements, but it can be explained mostly by the verbal remarks of several participants, who reported that they did see the trick mechanism, but being explicitly asked about the factuality of their account made them suspicious and think that question was as a “trick question.”

Undeceived individuals were also more confident about the correctness of their statement. After the first presentation, the median of their self-assessment on a one-to-seven point Likert scale was six whereas for deceived individuals, it was four, *U*_(_*_N_*_=120)_ = 706, *Z* = -5.75, *p* < 0.001. After the second presentation the median was seven for the undeceived individuals, and four for the deceived individuals, *U*_(_*_N_*_=120)_ = 127, *Z* = -6.61, *p* < 0.001.

Analysis of deception rates (see **Table 1**) during the first presentation show a strong influence of experimental condition, χ^2^_(4, *N* = 120)_ = 22.94, *p* < 0.001. Even the least effective misdirection technique (sideward gaze) differs clearly from the control condition, χ^2^_(1, *N* = 48)_ = 8.08, *p* = 0.004. The influence of experimental condition is maintained throughout the second presentation, χ^2^_(4, *N* = 120)_ = 11.43, *p* = 0.022. As predicted, the combined deception rates differ sharply between first (43.3%) and second presentation (12.5%), χ^2^_(*N* = 120)_ = 33.23, *p* < 0.001. (This χ^2^ statistic is the result of a McNemar’s test; all other χ^2^ statistics in this section are the results of Pearson’s tests).

**Table 1:** Number of deceived participants per experimental condition.

	Control		Eye contact		Red ball		Sideward gaze		Ball & gaze		Total	
	*N*	%	*N*	%	*N*	%	*N*	%	*N*	%	*N*	%
First presentation	1	4.2	13	54.2	13	54.2	9	37.5	16	66.7	52	43.3
Second presentation	0	0	1	4.2	4	16.7	3	12.5	7	29.2	15	12.5

Nothing indicated that the two videos making up each experimental condition were differently effective in misdirecting participants’ covert attention. All pairs of videos (except one) either exhibited equal deception rates or differed only by one deceived participant. This is true for both presentations. The biggest difference was observed after the first presentation of the Ball & Gaze condition, where one video deceived seven participants and the other video deceived nine. This difference fails to reach statistical significance even without the appropriate alpha adjustment, χ^2^_(1, *N* = 24)_ = 0.75, *p* = 0.386, suggesting no relevant differences between the videos used in each experimental condition.

No overall difference in deception rates between males and females was found for the first, χ^2^_(1, *N* = 120)_ = 1.22, *p* = 0.269, and second presentation, χ^2^_(1, *N* = 120)_ = 0.69, *p* = 0.408, of the video. Considering possible sex differences when orienting attention in response to social cues (Bayliss et al., 2005), only those conditions in which social misdirection was employed (Eye Contact, Sideward Gaze, and Ball & Gaze) were examined jointly. Again no differences were found for the first presentation, χ^2^_(1, *N* = 72)_ = 0.89, *p* = 0.345, and second presentation, χ^2^_(1, *N* = 72)_ = 0.11, *p* = 0.743, of the video.

Deceived and undeceived individuals (measured after the first presentation) did not differ regarding their self-reported previous experience with magic tricks. On a one-to-seven point Likert scale, the median of their self-assessment was two for both groups, *U*_(_*_N_*_=120)_ = 1596, *Z* = -0.94, *p* = 0.346. Scores ranged from one to seven, although only one individual reported having a lot of previous experience (seven).

### Amazement

Ratings of the questionnaire for the assessment of a deception demonstration were subject to a factor analysis using the maximum likelihood extraction method and varimax rotation. Four factors with eigenvalues greater than one, which together explained 66.9% of total variance, were extracted. Considering the individual items they represent (for a list of items, see Hergovich, 2004), the factors were named “amazement” (explaining 28.9% of total variance), “miracle” (explaining 16.7%), “fraud” (explaining 12.1%), and “no explanation” (explaining 9.2%). After reversing items with negative factor loadings, all items were grouped according to the factors on which they primarily loaded. Internal consistency for each grouping was examined using Cronbach’s α: 0.85 for “amazement,” 0.69 for “miracle,” 0.69 for “fraud,” and 0.60 for “no explanation.”

A binary logistic regression analysis was conducted, to examine the ability of the questionnaire and its four item groupings to predict (or, rather, correctly indicate) whether participants were able to detect the trick mechanism during the first presentation of the video. The model reached statistical significance, χ^2^_(4)_ = 34.7, *p* < 0.001, explained 33.7% (Nagelkerke’s *R*^2^) of the variance in deception rates and correctly identified the content (as categorized by the judges) of 73.3% of participants’ statements. Amazement was the only factor to make a significant contribution to the model, *B* = -0.80, *SE* = 0.19, Wald_(1)_= 17.80, *p* < 0.001.

**FIGURE 3:**
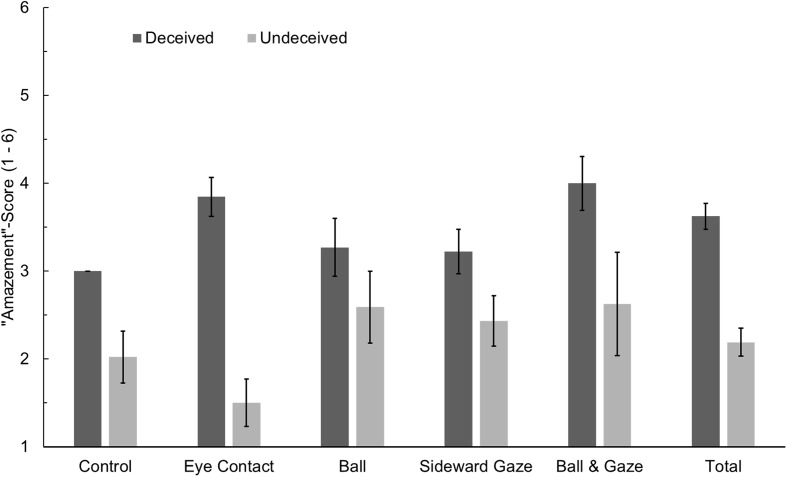
**“Amazement” scores for individuals who were deceived or undeceived during the first presentation of the video in the five experimental conditions.** Error bars denote standard errors of the mean. Note that only one participant was deceived in the control group (see **Table 1**).

Individuals who were undeceived after the first presentation had an average amazement score (on a one-to-six point Likert scale) of 2.19 (*SD* = 1.32) whereas those who were deceived had an average score of 3.63 (*SD* = 1.07), *t*_(118)_ = 6.38, *p* < 0.001. That most participants who were deceived after the first presentation were able to detect the trick mechanism during the second presentation, thus being undeceived when filling out the questionnaire, is worth noting. The mean amazement score for the control group was 2.06 (*SD* = 1.40); for the Eye Contact group, it was 2.77 (*SD* = 1.45); for the Red Ball group, it was 2.96 (*SD* = 1.29); for the Sideward Gaze group, it was 2.73 (*SD* = 1.05); and for the Ball & Gaze group, it was 3.54 (*SD* = 1.50), resembling the deception rates presented in **Table 1**. When analyzed using a two-way analysis of variance (ANOVA) with the factors experimental condition and deception (deceived vs. undeceived), no main effect of experimental condition, *F*_(4, 110)_ = 0.96, *p* = 0.431, $\eta_{p}^{2}$ = 0.034, and no interaction could be found, *F*_(4, 110)_ = 1.80, *p* = 0.134, $\eta_{p}^{2}$ = 0.061. However, a significant main effect of deception, *F*_(1, 110)_ = 15.08, *p* < 0.001, $\eta_{p}^{2}$ = 0.121, showed that group differences in amazement scores can be reduced to differential misdirection efficacy of the experimental conditions (see **Figure 3**).

### Gaze Data

**FIGURE 4:**
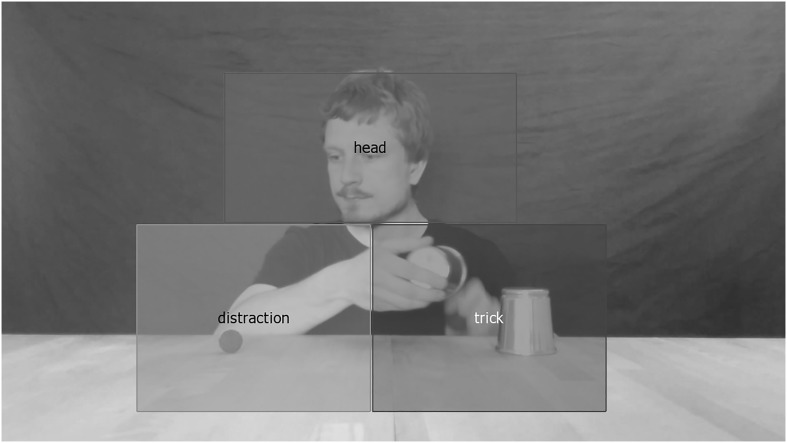
**Position and size of the three areas of interest (AOIs) relative to the performer, the cups, and the balls.** The scene chosen shows the moment in which the green ball is being loaded (in the trick AOI), while the performer directs his gaze (in the head AOI) toward the red ball (in the distraction AOI).

To analyze the eye-tracking data recorded during the presentations of the videos, three static, rectangular areas of interest (AOIs), each covering 11.3% (200,000 pixels^2^) of the total image area, were defined in such a way that, regardless of the video presented, all relevant objects, hand movements, and the head of the performer would be enclosed by them at all times (see **Figure 4**).

The AOI called “distraction” covered the area toward which, in the Ball, Sideward Gaze, and Ball & Gaze conditions, attempts of misdirection of the viewers’ attention were undertaken. The AOI called “head” covered the performer’s head in all conditions, although only in the Eye Contact, Sideward Gaze, and Ball & Gaze conditions, attempts of social misdirection were undertaken. The AOI called “trick” covered the area in which the to-be-discovered loading of the green ball happened, which occurred the same way in all conditions.

Viewers’ visual fixations were then categorized according to the AOI associated with their coordinates. From that their net dwell time, defined as the “sum of sample durations for all gaze data samples that hit the AOI” (SensoMotoric Instruments GmbH, 2014, p. 255), for the relevant time range was calculated. Analysis of eye-tracking data was restricted to the 19-to-20-s period of the video because in all the videos, attempts of misdirection and the loading of the green ball took place at the beginning of this time window.

A one-way multivariate analysis of variance (MANOVA) for viewers’ net dwell time on the three AOIs during the 19-to-20-s period of the first presentation of the video with experimental condition as between-subject factor revealed significantly different viewing patterns based on the presented trick choreography, *F*_(12, 345)_ = 7.07, *p* < 0.001, Pillai’s Trace = 0.59, $\eta_{p}^{2}$ = 0.197. Three one-way ANOVAs for each separate AOI confirmed that this is true for the distraction AOI, *F*_(4, 115)_ = 11.88, *p* < 0.001, $\eta_{p}^{2}$ = 0.292; the head AOI, *F*_(4, 115)_ = 10.09, *p* < 0.001, $\eta_{p}^{2}$ = 0.260; and the trick AOI, *F*_(4, 115)_ = 20.60, *p* < 0.001, $\eta_{p}^{2}$ = 0.417.

Nothing indicated that the two videos making up each experimental condition were influencing participants’ overt attention differently. Planned contrasts among the five pairs of videos for each of the three AOIs did not reveal statistically significant differences during the first presentation of the video. This finding is true for the 19-to-20-s period as well as for the total duration of the videos, which suggests similar viewing patterns for the paired choreographies.

**FIGURE 5:**
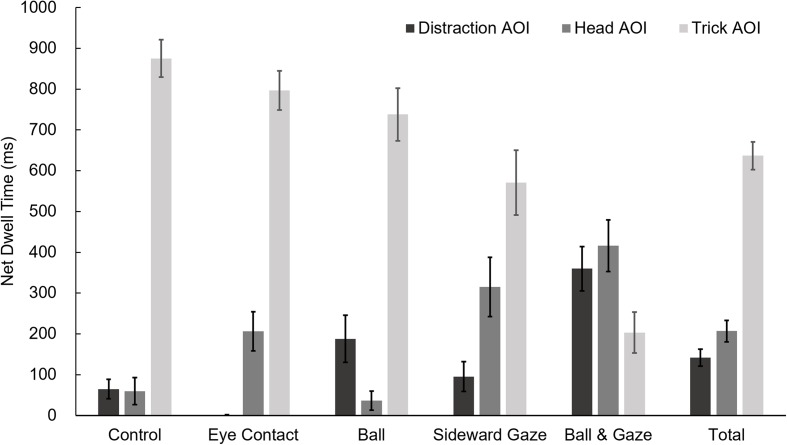
**Participants’ net dwell times on the three AOIs (see **Figure 4**) in the five experimental conditions during the 19-to-20-s period of the first presentation of the videos.** Error bars denote standard errors of the mean. Note that in the Eye Contact group, only two persons looked at the distraction AOI during the relevant timeframe for a few milliseconds each. In the Eye Contact condition (as well as in the control condition) no attempt of misdirection in the direction of the distraction AOI was made. When interpreting dwell times for the head AOI, be aware that in the Control and Ball conditions no social cues were employed.

Assigning the letters *A* to *E* to the experimental conditions in the order they appear in **Figure 5**, the following pairings achieved statistical significance (*p* < 0.05) in Games-Howell tests for each of the AOIs: distraction AOI, A < E, B < C, B < E, D < E; head AOI, A < D, A < E, B > C, B < E, C < D, C < E; and trick AOI, A > D, A > E, B > E, C > E, D > E. One-tailed Dunnett’s tests comparing experimental conditions to the control condition in the direction predicted by our hypotheses provided the same results.

More generally, in those experimental conditions in which an attempt of misdirecting viewers’ attention toward the distraction AOI was made (Ball, Sideward Gaze, and Ball & Gaze), we found that their net dwell time on the distraction AOI during the trick event was indeed longer (*M* = 214 ms) than in the other conditions (*M* = 33 ms), *t*_(118)_ = 4.55, *p* < 0.001 (one-tailed). This result was also true for the second presentation of the video, *t*_(118)_ = 3.00, *p* = 0.002 (one-tailed).

Also, in those experimental conditions in which an attempt of social misdirection was made (Eye Contact, Sideward Gaze, and Ball & Gaze), viewers’ net dwell time on the head AOI during the trick event was longer (*M* = 313 ms) than in the other conditions (*M* = 48 ms), *t*_(118)_ = 5.52, *p* < 0.001 (one-tailed). This result was also true for the second presentation of the video, *t*_(118)_ = 3.66, *p* < 0.001 (one-tailed).

Mixed design ANOVAs with experimental group as the between-subject factor and performance (first vs. second presentation of the video) as the within-subject factor revealed that only for the head AOI net dwell times in the 19-to-20-s period differed statistically significantly between first (*M* = 207 ms) and second presentation (*M* = 142 ms). A main effect of presentation was found, *F*_(1, 115)_ = 5.58, *p* = 0.020, $\eta_{p}^{2}$ = 0.046. No interaction between presentation and experimental condition was indicated, *F*_(4, 115)_ = 1.29, *p* = 0.279, $\eta_{p}^{2}$ = 0.043, but, of course, a strong main effect of experimental condition was found, *F*_(4, 115)_ = 11.88, *p* < 0.001, $\eta_{p}^{2}$ = 0.292.

**FIGURE 6:**
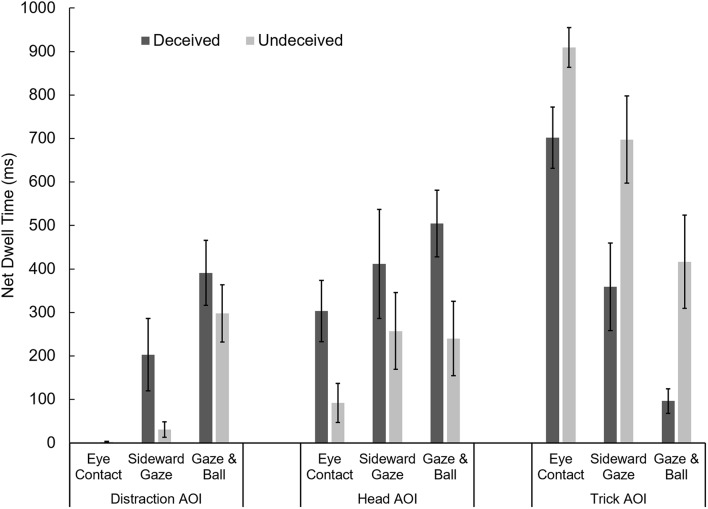
**Deceived and undeceived individuals’ net dwell time on the three AOI during the 19-to-20-s period of the first presentation of the videos in the three experimental conditions in which social misdirection was employed.** Error bars denote standard errors of the mean. As noted in **Figure 5**, participants did not orient their gaze toward the distraction AOI in the Eye Contact condition.

To further investigate the influence of social misdirection on the viewing patterns of deceived and undeceived individuals, those experimental conditions in which an attempt of social misdirection was made (Eye Contact, Sideward Gaze, and Ball & Gaze) were included into a two-way MANOVA with the factors “experimental condition” and “deception” (see **Figure 6**). The omnibus MANOVA revealed statistically significant main effects for experimental condition, *F*_(6, 130)_ = 8.55, *p* < 0.001, Pillai’s Trace = 0.57, $\eta_{p}^{2}$ = 0.283, and deception, *F*_(3, 64)_ = 7.57, *p* < 0.001, Pillai’s Trace = 0.26, $\eta_{p}^{2}$ = 0.262, but no interaction, *F*_(6, 130)_ = 1.21, *p* = 0.306, Pillai’s Trace = 0.11, $\eta_{p}^{2}$ = 0.053.

In those experimental conditions in which an attempt of misdirecting viewers’ attention toward the distraction AOI was made, the net dwell time on the distraction AOI (during the trick event of the first presentation of the video) of deceived individuals (*M* = 279 ms) was longer than that of undeceived individuals (*M* = 142 ms), *t*_(70)_ = 2.24, *p* = 0.014 (one-tailed).

As shown by the preceding ANOVA, the same is true for the head AOI in experimental conditions in which an attempt of social misdirection was made. The net dwell time on the head AOI of deceived individuals (*M* = 414 ms) was longer than that of undeceived individuals (*M* = 200 ms). Deceived individuals were also found to allocate overt attention on the trick AOI to a lesser extent (*M* = 484 ms) than were undeceived individuals (*M* = 754 ms), *t*_(118)_ = 4.22, *p* < 0.001 (one-tailed).

This analysis was extended to include the second presentation of the video. For the net dwell times on each AOI in the 19-to-20-s period a mixed-design ANOVA, with deception (deceived vs. undeceived) as the between-subject factor and performance (first vs. second presentation of the video) as the within-subject factor, was conducted. Only those experimental conditions in which an attempt of lateral misdirection toward the distraction AOI was made were included into the analysis of the net dwell time on the distraction AOI. A main effect of deception, *F*_(1, 70)_ = 11.16, *p* = 0.001, $\eta_{p}^{2}$ = 0.137; no main effect of performance, *F*_(1, 70)_ = 1.16, *p* = .286, η^2^_p_ = .016; and no interaction, *F*_(1, 70)_ = 0.52, *p* = 0.473, $\eta_{p}^{2}$ = 0.007, were found, reflecting a longer net dwell time for deceived individuals on both occasions and a general increase in net dwell time during the second presentation.

Again, for the analysis of the head AOI only those experimental conditions in which an attempt of social misdirection was made were examined. A main effect of deception, *F*_(1, 70)_ = 4.52, *p* = 0.037, $\eta_{p}^{2}$ = 0.061; a main effect of performance, *F*_(1, 70)_ = 6.27, *p* = 0.015, $\eta_{p}^{2}$ = 0.082; and an interaction, *F*_(1, 70)_ = 5.90, *p* = 0.018, $\eta_{p}^{2}$ = 0.078, were found, demonstrating a change in allocation of overt attention for those individuals who were deceived the first time they watched the video. The initial difference in net dwell time means between deceived and undeceived individuals (414 and 212 ms, respectively) completely disappears during the second presentation (200 and 197 ms, respectively).

The analysis of the trick AOI across all conditions revealed a main effect of deception, *F*_(1, 118)_ = 15.09, *p* < 0.001, $\eta_{p}^{2}$ = 0.113; a main effect of performance, *F*_(1, 118)_ = 4.46, *p* = 0.037, $\eta_{p}^{2}$ = 0.036; and no interaction, *F*_(1, 118)_ = 3.11, *p* = 0.081, $\eta_{p}^{2}$ = 0.026, reflecting longer net dwell time of undeceived individuals on both occasions.

Finally, whether deceived individuals may exhibit a general tendency to allocate overt attention on the performer’s face, irrespective of attempts of social misdirection [as implied by Tachibana and Kawabata (2014)], was investigated. For this purpose, only the first 16 s of the videos were included into the analysis. Because all movements of the performer that differ between experimental conditions begin approximately at the 18-s mark, the first 16 s of each video are highly comparable.

A mixed-design ANOVA with deception (deceived vs. undeceived) as the between-subject factor and performance (first vs. second) as the within-subject factor was performed. A main effect of performance, *F*_(1, 118)_ = 5.53, *p* = 0.020, $\eta_{p}^{2}$ = 0.045, which was caused by the group of deceived individuals, and a clear interaction between performance and deception, *F*_(1, 118)_ = 12.27, *p* = 0.001, $\eta_{p}^{2}$ = 0.094, were found. No main effect of deception could be established, *F*_(1, 118)_ = 0.04, *p* = 0.847, $\eta_{p}^{2}$ < 0.001.

Deceived individuals looked at the performer’s face for 24.4% of the analyzed timeframe (*M* = 3.91 s, *SD* = 2.81 s). Undeceived individuals looked at the performer’s face for 19.8% of the analyzed timeframe (*M* = 3.17 s, *SD* = 1.73 s). This difference barely achieved statistical significance when allowing for one-tailed testing, *t*_(118)_ = 1.77, *p* = 0.040.

Further exploration of the gaze data again revealed that deceived individuals compensated for their inability to detect the trick mechanism during the first presentation by looking at the performer’s face to a lesser extent during the first 16 s of the second presentation of the video, *t*_(51)_ = 3.19, *p* = 0.002 (paired samples, two-tailed), even during a time when no social cues are visible. Deceived individuals now looked at the performer’s face for 16.2% of the analyzed timeframe (*M* = 2.59 s, *SD* = 1.22 s) whereas undeceived individuals looked at the performer’s face for 21.5% of the analyzed timeframe (*M* = 3.43 s, *SD* = 1.68 s), *t*_(118)_ = 3.18, *p* = 0.002 (two-tailed).

## Discussion

### Deception

All the applied techniques of attentional misdirection were shown to be effective in diverting participants’ covert attention, by comparing the number of participants being unable to describe the mechanism of the trick in each group to the control condition. Because participants were allowed to answer the open question about the nature of the trick mechanism in any way they wanted, their statements ranged from accurate descriptions of the actual events to wild guesses about what could have happened. However, only a few statements lay somewhere between those extremes and were not obviously sortable, which is represented by very high interrater reliability. This is supported by significant differences in confidence between participants categorized as deceived or undeceived.

Unlike researchers of similar experiments (Kuhn et al., 2008b; Kuhn and Findlay, 2010), we found that forcing participants to specify whether their statements represent actual perceptions did not help in assessing the misdirection of covert attention at the time of the trick event. In a large number of cases, this direction led participants to become doubtful of their statements and to question the correctness of their memories. In addition, the nature of the experiment made them suspect to have been deceived without knowing it. Conversely, in a few cases, people also stated that they perceived false events (e.g., indications for video editing or the hand of an alleged accomplice). That such falsely positive statements did not occur in the previously mentioned experiments may be explained by their smaller sample size.

Distribution of deception rates confirms our hypothesis that all forms of misdirection, social and physical, were effective in distracting viewers’ covert attention, thereby confirming and extending previous research regarding the relevance of social misdirection. In particular, pure methods of social misdirection, in the form of either eye contact or sideward gaze, were sufficiently diverting to positively cause higher deception rates than in the socially neutral control condition. In previous research (Kuhn and Tatler, 2005; Tachibana and Kawabata, 2014), social and physical forms of misdirection were clearly confounded, or, when conceptually separated (e.g., Kuhn et al., 2009), the social cue was not the only misdirecting feature.

At least in this instance, pure social cues and visually salient objects did not seem to differ, regarding their utility in misdirecting covert attention. Certainly one can expect this result to vary depending on the trick choreography and the specific stimulus used. We could, however, show that in this study, social misdirection was not just a useful complement to a more elaborate choreography, but was in itself sufficiently deceptive to be comparable to the surprising occurrence of a red ball. Even the entirely uninformative sideward gaze toward the empty table surface seemed to be comparable to the red ball in its misdirection effectiveness.

Although, as expected, the combination of sideward gaze and red ball seemed to result in the highest deception rates, such differences between experimental conditions did not reach statistical significance. Because of the dichotomous nature of the deception measure, a further increase in sample size or a sharp reduction in experimental conditions would be necessary for ranking misdirection techniques according to their respective effectiveness.

As predicted, deception rates dropped sharply after the second presentation of the performance. This seemed to be true in all conditions; nevertheless, they did not reach 0% as in the experiments of Kuhn and Tatler (2005) or Kuhn et al. (2008b). Descriptive examination suggested the effect of the seemingly most deceptive experimental condition (Ball & Gaze) to be the most persistent because about half of the participants who remained deceived after the second presentation belonged to this group.

Reported previous experience with magic tricks was not found to differ between deceived and undeceived individuals. This is not particularly surprising, as our sample consisted of average university students and staff, only one of which reported having a lot of previous experience with magic tricks, suggesting the absence of experts in the field.

No sex differences in deception rates were found, even when only considering experimental conditions in which social cues were employed. However, our sample size of 120 participants would only have been adequate to detect a difference under the assumption of a large effect size (Oyeyemi et al., 2010). Individual differences identifiable through reaction time paradigms are likely hard to replicate using complex, life-like stimulus material and a dichotomous dependent variable, such as the perception of a singular event is.

### Amazement

Amazement was found to be the factor explaining the most variance in the answers to the questionnaire for the assessment of a deception demonstration. This factor was also best suited to predict whether a participant had been deceived during the first presentation of the trick. Although amazement scores varied between experimental conditions, these variations were shown to be the result of their relative effectiveness in misdirecting viewers’ covert attention.

The concept of amazement can be regarded as the phenomenological opposite of the “aha! experience,” investigated by Danek et al. (2014). Whereas aha! experiences accompany the insightful solution of a difficult problem and can be triggered by discovering the mechanism of a magic trick, amazement stems from the initial failure to do so. Parris et al. (2009) explored the neural correlates of violations of expected causal relationships in the context of magic tricks, using fMRI. We consider further elaborating on the phenomenological characteristics of the concept of amazement, and, more generally, the subjective experiences elicited by the observation of magic tricks, in future studies to be worthwhile.

Our findings suggest that a more comprehensive questionnaire focusing on the measurement of participants’ amazement after viewing a magic performance might be used as an indirect measure of perception of a trick event in situations, where open questions, which require subsequent rating by judges, or forced-choice paradigms are not feasible or desirable.

For the sake of optimal comparability of first and second presentation of the video, only the short questions pertaining to the trick mechanism were asked in between both presentations, while the amazement-specific items of the questionnaire were presented at the end of the experiment. This means that when answering these items, most of the initially deceived individuals by then had discovered the trick mechanism. Undoubtedly questioning participants immediately after having seen the performance would lead to increased discriminatory power in future experiments.

### Overt Attention

Although for the period preceding the trick event no significant differences in participants’ allocation of overt attention were found between the presented videos, during the trick event, highly significant differences between experimental conditions were observed for all the examined AOIs.

As predicted, when lateral misdirection was employed, either through social or physical triggers, participants spent more time looking at the area emphasized by the misdirection during the trick event of both presentations. Of all the experimental conditions, lateral misdirection was the strongest for the combination of social and physical triggers (Ball & Gaze). All comparisons to other conditions, except the condition in which only a physical trigger was employed (Ball), reached statistical significance. Interestingly, when it was not reinforced by a gaze cue, the red ball was not distracting enough to cause a significant difference from the control condition. This again demonstrates the relevance of social misdirection in further strengthening a physical misdirection technique (e.g., Kuhn et al., 2008b).

As predicted, when social misdirection was employed, through either eye contact or a sideward gaze, participants spent more time looking at the area of the performer’s head during the trick event of both presentations. Although this was true when analyzing the aggregate results, some comparisons between individual groups failed to reach statistical significance. Our prediction that participants would spend more time looking at the head of the performer when he made eye contact than in the control condition was such a case. Watching the recordings of the fixations of participants who reciprocated eye contact reveals that they only did so very briefly.

Interestingly, the combination of social and physical triggers (Ball & Gaze) also seemed to be most effective in attracting overt attention to the head, significantly more than making eye contact was. An explanation for this finding, supported by studying replays of individual gaze recordings, is that some participants first oriented overt attention toward the performer’s moving head and afterward toward the cued at location. Because such a viewing pattern requires at least two fixations and one saccade, this strategy of misdirection of overt attention seems to be doubly effective.

Also, when a sideward gaze was employed the distinct movement of the performer’s head not only worked as a lateral gaze cue, but was in itself more attentionally compelling than making eye contact. This result, of course, poses the question of whether the social aspect of the cue or the increased visual salience of the moving head caused the misdirection. However, all social cues (except in still images) probably involve movement and are therefore more visually salient than other image areas. In addition to being visually salient, the sideward cue may have corresponded better to a priori expectations participants had about the trick mechanism. In the Eye Contact group disengagement of attention from the performer’s head may have been quicker due to the missing contextual relevance of his gaze.

Superior capability of the Ball & Gaze condition to misdirect overt attention was also reflected by the time spent looking at the area in which the trick event happened, which was significantly lower than in all other conditions. Although the eye-tracking data of the control condition suggest a close coupling of overt and covert attention in the absence of misdirection, in the case of the Eye Contact group, only a tiny, statistically non-significant decrease in average time spent looking at the area of the trick event was sufficient to entail a massive increase in deception rates.

However, strong evidence for a link between overt and covert attention was found when the overall viewing patterns of deceived and undeceived individuals at the time of the trick event were compared. When social cues were employed, deceived individuals clearly spent more time looking at the head of the performer and less time looking at the area of the trick event (**Figure 6**). An overall reduction in time spent looking at the performer’s head during the trick event was noticeable between first and second presentation across all conditions. This reduced time spent looking at the performer’s head was accompanied by an increase in time spent looking at the area of the trick event, which was generally higher for undeceived individuals, reflecting the observed decrease in deception rates.

By more closely examining those conditions under which social misdirection was employed, we found an interesting change in viewing strategies of deceived individuals. Although they initially spent about twice as much time looking at the head of the performer than undeceived individuals did, they compensated for their failure in detecting the trick mechanism by matching their viewing behavior to that of undeceived individuals. This change in viewing strategy can even be observed in the absence of social cues, during the initial period of the performance, when there were no misdirection attempts.

Although Tatler and Kuhn (2007) also report generally decreased overt attention to the performer’s head during the second presentation of a magic performance, we found this reduction to be attributable to the subgroup of initially deceived participants. This finding suggests a top-down modulation of overt attention allocation in response to task demands already observed by Kuhn et al. (2008b). They reported prior information about a trick to decrease deception rates and influence gaze patterns. In our experiment participants may have disengaged attention from image areas (the face of the performer) they had previously learned to be uninformative or misleading.

The tendency of viewers who have a lower probability of discovering a trick mechanism to preferentially allocate overt attention to the head has already been described by Tachibana and Kawabata (2014), although they only analyzed a period in which an act of social misdirection actually happened. Because of the coupling between overt and covert attention (Kuhn and Findlay, 2010), that looking at a certain image area decreases the probability of perceiving an event happening in a different area is expected. Beyond that, we found indications that individuals may generally differ in their viewing preferences, which alters their chances of being deceived. However, the effect could also have been an artifact of a person’s central fixation bias (Tatler, 2007). Future research may be able to uncover further factors that influence the likelihood of deception while pushing the limits of what is possible within the constraints of the laboratory.

## Author Contributions

All authors listed, have made substantial, direct and intellectual contribution to the work, and approved it for publication.

## Conflict of Interest Statement

The authors declare that the research was conducted in the absence of any commercial or financial relationships that could be construed as a potential conflict of interest.
